# Spatial and temporal patterns and factors associated with mortality from Chagas’ disease: an ecological study, Ceará, 2002-2022

**DOI:** 10.1590/S2237-96222025v34e20240852.en

**Published:** 2025-08-08

**Authors:** Lara Lídia Ventura Damasceno, George Jó Bezerra Sousa, Thiago Santos Garces, Virna Ribeiro Feitosa Cestari, Jéssica Cristina Moraes de Araújo, Thereza Maria Magalhães Moreira, Maria Lúcia Duarte Pereira

**Affiliations:** 1 Universidade Estadual do Ceará, Programa de Pós-Graduação Cuidados Clínicos em Enfermagem e Saúde, Fortaleza, CE, Brasil; 2 Ministério da Saúde, Brasília, DF, Brazil; 3 Universidade Estadual do Ceará, Programa de Pós-Graduação em Saúde Coletiva, Fortaleza, CE, Brazil

**Keywords:** Chagas’ disease, Mortality, Demographic Indicators, Spatial Analysis, Ecological Studies, Enfermedad de Chagas, Mortalidad, Indicadores Demográficos, Análisis Espacial, Estudios Ecológicos

## Abstract

**Objective:**

To describe the temporal and spatial pattern of mortality from Chagas’ disease in Ceará in the period 2002-2022.

**Methods:**

This is an ecological study, which considered Ceará, its health macro-regions and municipalities as the units of analysis, based on secondary data from the Mortality Information System. Temporal analysis was performed to calculate the annual percentage variation, as well as the spatial autocorrelation indicators (global and local Moran index, Getis-Ord Gi* and scanning). The indicators were inserted into non-spatial and spatial regression models.

**Results:**

1,041 deaths were reported, with an average mortality rate of 0.57 deaths/100,000 inhabitants. Cariri (35.45%) and Litoral Leste (the East Coast) (23.05%) stood out in notifications, while the Litoral Norte (North Coast) region had a higher mortality rate (2.15/100 thousand inhabitants). There was a significant increase in mortality rates from the disease on the Litoral Leste and in the Sertão Central (Central Hinterlands). High coefficients were observed for the Gini indices (9.6; p-value<0.001) and municipal human development (17.1; p-value<0.001), which suggested that areas with greater income inequality and lower human development indices are impacted to a greater extent by mortality from Chagas’ disease. Areas of high mortality tend to be close to other areas with high mortality rates.

**Conclusion:**

Areas of greater risk were identified, especially in the Litoral Leste and Sertão Central macro-regions, while other regions showed stability throughout the period. Spatial models pointed to influence from factors such as income inequality and human development.

## Resumen

**Objetivo:**

Describir el patrón temporal y espacial de la mortalidad por enfermedad de Chagas en Ceará en el período 2002-2022.

**Métodos:**

Se trata de un estudio ecológico, que consideró como unidades de análisis a Ceará, sus macrorregiones de salud y municipios, con base en datos secundarios del Sistema de Información de Mortalidad. Se realizó un análisis temporal para calcular la variación porcentual anual, así como los indicadores de autocorrelación espacial (índice de Moran global y local, Getis-Ord Gi* y scan scan). Los indicadores se insertaron en modelos de regresión espacial y no espacial.

**Resultados:**

Se reportaron 1.041 defunciones, con una tasa de mortalidad promedio de 0,57 defunciones/100.000 habitantes. Cariri (35,45%) y Litoral Este (23,05%) se destacaron en las notificaciones, mientras que el Litoral Norte presentó mayor tasa de mortalidad (2,15/100 mil habitantes). Se observó un aumento significativo de las tasas de mortalidad por la enfermedad en la Costa Este y en el Sertão Central. Se observaron coeficientes altos para el índice de Gini (9,6; valor p<0,001) y el índice de desarrollo humano municipal (17,1; valor p<0,001), lo que sugiere que las zonas con mayor desigualdad de ingresos y menores índices de desarrollo humano se ven afectadas en mayor medida por la mortalidad por enfermedad de Chagas. Las zonas de alta mortalidad tienden a estar cerca de otras zonas con altas tasas de mortalidad.

**Conclusión:**

Se identificaron áreas de mayor riesgo, especialmente en las macrorregiones Litoral Este y Sertão Central, mientras que las demás regiones mostraron estabilidad a lo largo del período. Los modelos espaciales apuntaron a la influencia de factores como la desigualdad de ingresos y el desarrollo humano.

**Ethical aspects**

This research used public domain data and anonymized databases.

## Introduction

Chagas’ disease affects 6 to 7 million people globally, while being responsible for 200,000 deaths annually (1). Brazil, a country with a tropical and subtropical climate, has high rates of prevalence and mortality from the disease in Latin America. The country accounts for more than 10,000 deaths per year, 1 million cases and 25 million people at risk of infection, which reinforces the disease’s endemicity pattern in Latin America. Among Brazilian regions, the Northeast plays a relevant role in the national epidemiology of Chagas’ disease, given the confluence of morphoclimatic factors of the caatinga, prevalence of precarious human housing such as mud houses, with peridomestic environments susceptible to the presence of the vector, in addition to the predominance of populations in social vulnerability and with limited access to health care (2-3).

Ceará is one of the priority areas of action of the Healthy Brazil Program, launched in 2024 by the Ministry of Health, to eliminate socially determined diseases, including Chagas’ disease (4). This list of diseases disproportionately affects poor populations, closely relating to environmental, social, and economic aspects, while at the same time being historically marked by stigma, discrimination, and low economic attractiveness (5).

Considering georeferencing techniques as a technology to support research, management and healthcare assistance, the relevance of mapping the spatial and temporal characteristics of Chagas’ disease stands out. The influence of this disease on the health-disease relationship of populations is also highlighted, with a view to recognizing demands and building evidence for the management of effective, consistent, and sustainable public policies, based on the identification of elevated risk clusters (6). 

This study aimed to describe the temporal and spatial pattern of mortality from Chagas’ disease in Ceará in the period 2002-2022.

## Methods

### Study design

This is an ecological study that used geoprocessing and time series techniques. Secondary data from the Mortality Information System of the Information and Informatics Department of the Bazilian Unified Health System were considered.

### Context

The geographic area of Ceará, its health macro-regions and municipalities were considered as units of analysis. The state is in the Northeast region of Brazil and its capital is Fortaleza. The territorial extension is 148,894.447 km^2^, with an estimated population of 8,794,957 inhabitants; of these, it is estimated that at least 22.6% reside in rural areas, distributed in 184 municipalities and five health macro-regions: Fortaleza, Cariri, Litoral Leste, Sobral e Sertão Central. Health macroregions are composed of border municipalities, which share similar economic and social characteristics (7).

Ceará is home to a vast number of species of vectors of *Trypanosoma cruzi*, the etiological agent of Chagas’ disease. These are hematophagous insects, from the subfamily Triatominae, popularly known as “barbeiros.” Regarding the native species of the caatinga biome, in semi-arid areas, the following stand out: *Triatoma brasiliensis, Triatoma pseudomaculata, Triatoma sordida, Panstrongylus megistus, Panstrongylus lutzi* and *Rhodnius nasutus* are the most elevant in epidemiological terms (8).

### Data source

All death records reported in the Mortality Information System between January 2002 and December 2022 were considered, with the underlying cause being code B57 of the International Classification of Diseases (ICD-10), which includes: B570 – Acute Chagas’ disease, with heart involvement; B571 – Acute Chagas’ disease, without heart involvement; B572 – Chagas’ disease (chronic) with heart involvement; B573 – Chagas’ disease (chronic) with digestive system involvement; B574 – Chagas’ disease (chronic) with nervous system involvement; and B575 – Chagas’ disease (chronic) with involvement of other organs. The databases used to construct the results are available in the SciELO Data repository and are publicly accessible (9).

### Statistical methods

To calculate the crude mortality rate, the number of deaths from Chagas’ disease per year in Ceará was taken as a basis, divided by the denominator of the resident population in the state in that year, multiplied by the coefficient of 100 thousand inhabitants. Mortality rates were calculated for the variables sex, age group, race/skin color and education, considering the number of deaths and stratified population, multiplied by the coefficient of 100 thousand inhabitants.

In the time series analysis, the data were allocated to the Joinpoint software Regression Program, which identified the inflection points of the period, in addition to calculating the annual percentage variation, the mean annual percentage variation and the 95% confidence interval (95%CI). The year was defined as the independent variable and the mortality rate for each year as the dependent variable, assuming zero to two inflection points, according to the Monte Carlo permutation. For all analyses, first-order autocorrelation of errors was considered, while health data were dependent on previous events. Negative or positive values of annual percentage variation and mean annual percentage variation with statistical significance indicated time series with decreasing and increasing patterns. When there was no statistical significance, the series was considered stationary (10).

In the spatial analysis, the average mortality rate for each municipality (crude rate) was calculated, adopting as numerator the average number of deaths from Chagas’ disease for each municipality, divided by the population of the municipality in the middle of the period, corresponding to the year 2012, multiplied by the coefficient of 100 thousand inhabitants. To minimize instability, the crude rates were smoothed using the local empirical Bayesian method, which included the spatial effects of geographic neighbors (bordering municipalities), by assigning values to neighboring municipalities (1) and non-neighbors (0), using the spatial weight matrix (queen contiguity) (11).

The identification of spatial clusters was conducted using global and local autocorrelation methods, arranged by the global Moran index and the local Moran index (Lisa Map), respectively, by Getis-Ord Gi* and by scanning. Lisa Map helped measure the degree of spatial association of each municipality, through the identification of high-high, low-low, high-low and low-high patterns. The high-high and low-low patterns referred to areas with high or low mortality values and were clustered geographically, indicating a positive spatial association. The high-low and low-high patterns indicated a negative spatial association, that is, they represented municipalities with discrepant values in relation to their neighbors. Getis-Ord Gi* inferred agglomeration in areas with high rates (hot areas) and areas with low rates (cold areas), from the creation of Z scores (12).

The scanning was purely spatial and was used to identify clusters and calculate the relative risk for each municipality, adopting the discrete Poisson model. The following criteria were considered: maximum cluster size equal to 50.0% of the population at risk, circular clusters and 999 replications. Relative risk values >1 were seen in municipalities with a risk of death from Chagas’ disease higher than the country’s risk (13).

Some indicators were selected from the Brazilian Human Development Atlas to analyze the influence of socioeconomic indicators on mortality from Chagas’ disease in the municipalities of Ceará: Gini index, municipal human development index (MHDI), social vulnerability index, illiteracy rate among people over 18 years old, unemployment rate among people over 18 years old, percentage of people registered in the Single Registry for Social Programs who receive “Bolsa Família” (a federal financial benefit for poor families), percentage of people in households with inadequate water supply and sanitation, and percentage of people in households with walls that are not made of masonry or prepared wood.

Different spatial and non-spatial regression methods were used. The generalized linear model (GLM) was applied, due to the variability of mortality rates and the failure to meet the assumptions of simple linear regression. Then, spatial models such as spatial lag and spatial error were evaluated, to capture the spatial autocorrelation of the data, evaluated using the Rho parameters (spatial lag) and Lambda (spatial error). The fit of these models was compared using the Akaike information criterion, with lower values indicating better quality of fit. Finally, the geographically weighted regression (GWR) model was adjusted to identify local variations in the coefficients. The quality of the analysis was measured by the pseudo R^2^, which reflected the proportion of variability explained locally. All regression analyses were performed in R software (version 4.4.1) with the spdep, GWmodel, sf and spgwr packages.

After extration in CSV format, the data were tabulated in Microsoft Excel spreadsheets and imported into the software QGis, in which all maps were created. Bayesian statistics, spatial autocorrelation test and Getis-Ord Gi* technique were performed in the GeoDa software. The scanning technique and RR calculation were performed using the SaTScan software. All regressions with social development indicators were run using the R software.

## Results

1,041 deaths from Chagas’ disease were reported in Ceará in the period 2002-2022, equivalent to an average of 50 deaths/year. The highest mortality rates were recorded in men, aged 80 or over, of brown race/skin color, illiterate or with less than three years of education (Table 1).

**Table 1:** Sociodemographic characterization of deaths and standardized mortality rate for Chagas’ disease in the period 2002-2022. Ceará, 2024 (n=1.041)

Variable	Frequency	Mortality rate^a^
Gender		
Male	694	0.78
Female	347	0.37
**Age group** (years)		
0-4	1	0.01
5-9		
10-14		
15-19	1	0.01
20-29	6	0.02
30-39	41	0.15
40-49	112	0.50
50-59	206	1.31
60-69	230	2.16
70-79	224	3.54
80+	219	6.64
**Race/skin color**		
White	265	0.51
Black	53	0.42
Yellow	3	1.27
Brown	676	0.57
Indigenous	2	0.24
**Education** (years)		
None	350	0.19
1-3	302	0.16
4-7	144	0.08
8-11	46	0.03
12+	14	0.01

^a^Mortality rate calculated for the coefficient of 100 thousand inhabitants.

The state’s mortality rate was 0.57 deaths/100,000 inhabitants. The notifications were made, mostly, in the health macro-regions of Cariri (n=369; 35.35%; 1.21 deaths/100 thousand inhabitants) and Litoral Leste (n=240; 23.05%; 2.15 deaths/100 thousand inhabitants). The other macro-regions were Fortaleza (n=182; 17.48%; 0.19 deaths/100,000 inhabitants), Sobral (n=172; 16.52%; 0.51 deaths/100,000 inhabitants) and Sertão Central (n=78; 7.49%; 0.59 deaths/100,000 inhabitants).

The time series indicated a stationary trend for Ceará and in the macro-regions of Cariri, Sobral and Fortaleza. The East Coast showed significant annual growth of 2.03% (95%CI 0.11; 4.19). The Sertão Central macroregion showed a significant increase of 8.62% between 2002 and 2020 (95%CI 4.89; 25.08), when a stationary trend started to show (Table 2).

**Table 2:** Annual percentage variattion, mean annual percentage variation, inflection point and 95% confidence interval (95%CI) of mortality rates due to Chagas’ disease in the period 2002-2022. Ceará, 2024 (n=1.041)

	Annual percentage variation 1 (95%CI)	Inflection point	p-value	Annual percentage variation 2 (95%CI)	Average annual percentage variation (95%CI)	p-value
Ceará	0.90 (-0.19; 2.07)				0.90 (-0.19; 2.07)	
**Litoral Leste**/Jaguaribe	2.03^a^ (0.11; 4.19)		0.042		2.03^a^ (0.11; 4.19)	0.042
**Sertão Central**	8.62^a^ (4.89; 25.08)	2020	0.026	-58.51 (-81.73; 2.28)	-1.34 (-8.74; 7.42)	
Cariri	0.27 (-1.63; 2.31)				0.27 (-1.63; 2.31)	
Sobral	-0.19 (-2.73; 2.39)				-0.19 (-2.73; 2.39)	
Fortaleza	0.03 (-2.67; 2.90)				0.03 (-2.67; 2.90)	

**Figure 1:**
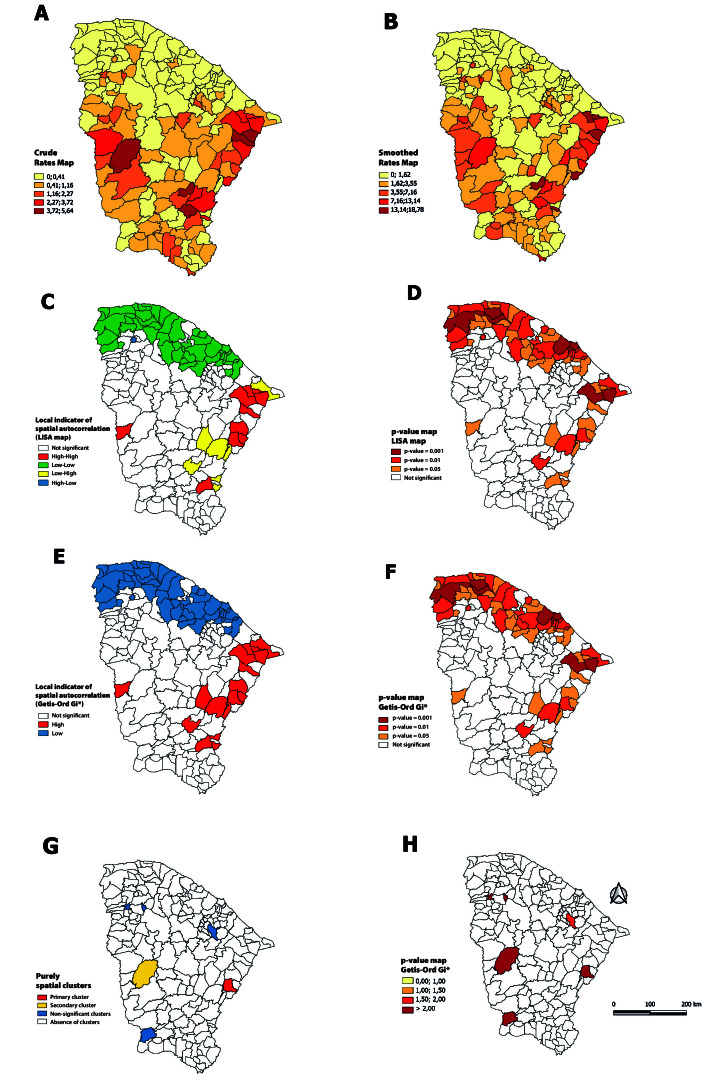
Spatial distribution of crude mortality rates from Chagas’ disease (A), rates smoothed by the local empirical Bayesian method (B), local indicator of spatial autocorrelation (C), respective statistical significance (D), local Getis-Ord Gi* autocorrelation indicator (E), respective statistical significance (F), purely spatial clusters identified by scanning (G) and municipal relative risk (H), in the period 2002-2022. Ceará, 2024 (n=1.041)

Spatial analyses confirmed the consistency of the results obtained by different methods, including the global Moran index (0.254), which indicated positive spatial autocorrelation. Crude mortality rates were irregularly distributed throughout the period (Figure 1A). When smoothed by the local empirical Bayesian method (Figure 1B), the highest rates were observed in the municipalities of Litoral Leste and Cariri. 

High-high patterns (Figure 1C), i.e., municipalities with high mortality rates surrounded by others with equally high rates, were observed in the localities of Russas, Palhano, Itaiçaba, Jaguaruana, Alto Santo, Iracema and Potiretama on the Litoral Leste; in Lavras da Mangabeira in Cariri; and in Novo Oriente in the Sobral region. The coastal area of the state that includes the Sobral and Fortaleza macro-regions showed a low-low pattern, which generally represented locations with low mortality rates for Chagas’ disease. 

High-low patterns could be seen in Aracati, Jaguaribe and Pereiro, on the Litoral Leste; in Iguatu, Umari and Ipaumirim, in the Cariri region; and in Solonópole, in Sertão Central. Only Alcântaras, located in the Cariri region, showed a low-high pattern. Both patterns revealed locations with different values in relation to their neighbors, which represented spatial discrepancies. The Getis-Ord Gi* technique confirmed hot areas in the Litoral Leste and Cariri regions and cold areas in Sobral and Fortaleza (Figure 1E). The scanning detected six spatial clusters (Figure 1G), with Iracema, a municipality on the Litoral Leste, being the primary cluster.

The regression models applied to the social development indicators associated with mortality from Chagas’ disease in Ceará were observed (Table 3). The GLM model presented the worst fit, which indicated that the spatial variation of the data was not well captured. The spatial lag model showed better adaptation to the data, which proved that the inclusion of spatial structure was fundamental to capture the spatial dependence between municipalities. The Gini coefficient in this model made clear that the effect of income inequality is less pronounced when spatial dependence is considered. The MHDI presented a coefficient close to the significance level. The spatial error model indicated spatial correlation in the residuals, suggesting that although the fit was good, there were dependencies not captured by the model. The GWR model presented a worse fit than the other spatial models, which indicated that the effects of the variables changed spatially in a non-uniform manner.

**Table 3:** Association between social development indicators and mortality from Chagas’ disease, according to regression models of generalized linear model (GLM), spatial lag, spatial error, and geographically weighted regression (GWR), in the period 2002-2022. Ceará, 2024 (n=1.041)

Indicator	GLM		Spatial error			Spatial lag			GWR	
Coefficient	EP^a^	Coefficient	EP^a^	p-value	Coefficient	EP^a^	p-value	Coefficient	EP^a^
Constant	16.63	<0.001	8.11	3.88	0.036	8.05	3.41	0.018	13.67	3.89
Gini	9.64	5.08	4.43	1.91	0.021	4.51	1.61	0.005	5.99	5,972
MHDI^b^	17,19	<0.001	6.78	4.42	0.125	7.41	3.92	0.059	8.43	14.60
IVS^c^	0.14	0.94	-	-	-	-	-	-	-	-
Illiteracy rate^a^	<0.001	0.65	-	-	-	-	-	-	-	-
Unemployment rate among people over 18 years old	0.03	0.28	-	-	-	-	-	-	-	-
Bolsa Família (Federal program)	<0.001	0.80	-	-	-	-	-	-	-	-
Households without water or sanitation	<0.001	0.67	-	-	-	-	-	-	-	-
Homes without masonry or wooden walls	0.01	0.30	-	-	-	-	-	-	-	-
Rho (spatial lag)^d^	-	-	-	-	-	0.55	0.07	1,181	-	-
Lambda (spatial error)^e^	-	-	0.55	0.07	1,571	-	-	-	-	-
R^2f^	0.14	-	0.32	-	-	0.32	-	-	0.09	-
Akaike information criterion^g^	540.72	-	500.29	-	-	497.26	-	-	528.17	-

^a^SE=standard error; ^b^MHDI=municipal human development index; ^c^IVS=social vulnerability index; ^d^Measures spatial dependence in the dependent variable and indicates the effect of the unit on its neighbors; ^e^Evaluates spatial correlation in model residuals, indicating dependence not captured by traditional regression; ^f^Represents the proportion of variance explained by the model, indicating its fit to the data. ^g^Measures model quality considering fit and complexity, being lower for more efficient models.

**Figure 2:**
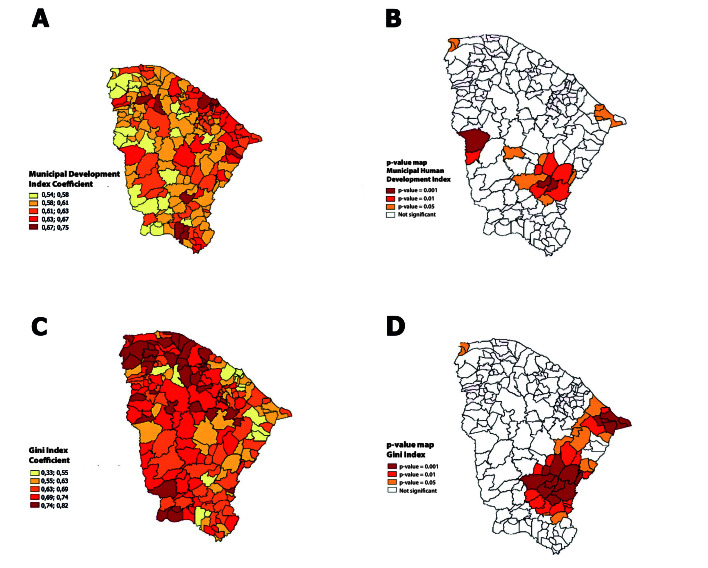
Geographically weighed spatial regression of the spatial distribution of the mortality rate from Chagas’ disease for the human development index (A), respective statistical significance (B), spatial distribution of the mortality rate from Chagas’ disease for the Gini index (C), respective statistical significance (D), in the period 2002-2022. Ceará, 2024 (n=1.041)

The spatial distribution of the coefficients estimated by the GWR for the social development indicators was highlighted (Figure 2 and Table 3). Regions such as Litoral Leste and Cariri presented more pronounced negative coefficients for the Gini, indicating a greater influence of income inequality in reducing mortality rates locally, while areas such as Sobral and Fortaleza showed smaller and non-significant effects. For the MHDI, the Litoral Norte and Sertão Central regions had positive and significant coefficients, which showed a greater impact of human development on mortality. These patterns reinforced the superiority of the spatial lag and spatial error models in capturing global spatial dependence and justified the usefulness of GWR to detect local variations, by highlighting the spatial complexity of these determinants in mortality from disease, complementing the global analysis of Chagas.

## Discussion

Mortality from Chagas’ disease in Ceará remained stable, with a prevalence profile among men, elderly people aged 80 and over, of brown and yellow race/skin color and with low education levels. The greatest occurrence was in the Litoral Leste and Cariri macro-regions, while Fortaleza and Sobral had a lesser impact. The relationship between socioeconomic indicators and mortality from Chagas’ disease varied regionally and highlighted income inequality and municipal human development as the most influential factors. These findings reinforced the persistence of Chagas’ disease as a public health problem in the state, especially in historically vulnerable areas.

As a limitation of this study, the use of secondary data stands out, as they were inaccurate in their records, due to inadequate filling of the system, in addition to underreporting. The professionals involved and responsible for the process of reporting deaths from Chagas’ disease were doctors, nurses, professionals from the Death Verification Service and the Forensic Medical Institute, epidemiologists, and health surveillance technicians, as well as professionals from the Mortality Information System. The results of the analysis should be interpreted with due caution, considering potential biases or inaccuracies in the data.

The epidemiological profile and geographic location of the event favored the interpretation that Chagas’ infection was related to worse outcomes, such as mortality, in residents and workers in rural areas, theoretically more exposed to the etiological agent of the disease (2).

Despite the predominance of cases in rural areas, it is worth noting that the macro-regions have other economic activities, such as commerce and crafts. Chagas’ disease and its outcomes, initially restricted to rural and less populated areas, have shown a tendency towards urbanization, favoring the transmission of the disease (14-15). These findings were similar to secondary data on mortality from neglected diseases in Brazil, including Chagas’ disease (16).

The ethnic profile identified demonstrated a significant association between brown and Black race/skin color and a worse prognosis for Chagas’ disease. This result must be analyzed with caution, as the northeastern population has a high degree of miscegenation, which makes accurate classification difficult.

The findings revealed that mortality from Chagas’ disease in Ceará is directly and inversely proportionally associated with the Gini index and the MHDI, respectively, that is, the greater the income inequality and the worse the living and development conditions, the higher the mortality rate from Chagas’ disease. This scenario is due to the considerable socioeconomic heterogeneity of the Brazilian Northeast region, recognized as the poorest in the country. These inequalities are also present in the health services/assistance component in access and continuity of care, especially in macro and micro regions with high vulnerability, such as Sertão Central and Litoral Leste (18). 

These regions are characterized by a concentration of extremely poor municipalities, represented by low MHDI and the Brazilian index of deprivation and access to health. Social inequality influences the search for better living conditions and, as a result, many individuals move to other regions and states. They often remain with a low standard of living, clustering in excluded territories lacking public services, including health care.

The Litoral Leste is marked by a lack of care of high and medium complexity, which motivated the investment in the Vale do Jaguaribe Regional Hospital, opened at the end of 2021 (19). Municipalities such as Russas and Jaguaruana, despite being densely populated commercial hubs, exhibit unresolved deficiencies in terms of the availability and viability of healthcare and strategies to combat poverty. The weaknesses were associated with geographic and transport characteristics, inadequate delimitation of the service coverage area, lack of government support and deficient distribution of resources (20-21). 

The Cariri region had a contingent of more than 400 thousand people living in extreme poverty and concentrated highly complex facilities in Barbalha, Brejo Santo, Crato and Juazeiro do Norte, in the extreme south of the state, with the Cariri Regional Hospital, opened in 2011, being the healthcare reference. The health authorities in the region, in cooperation with the Pan American Health Organization, are leading the “Open Arms” project, focusing on the decentralization and regionalization of health interventions, aiming at planning, and strengthening lines of care (22).

The North region, which has low-low standards, is based in Sobral and is recognized for its well-established economic situation, in addition to its educational hub, with an education system consistently ranked among the best in Ceará and Brazil. It is responsible for the highest density of health services in the region, with emphasis on the Hospital Regional Norte, Santa Casa de Sobral and Hospital do Coração. These are references in medium and high complexity procedures and play a vital role in healthcare in the region, acting as the main destination for referrals for emergencies and elective procedures (23). 

It is postulated that death from Chagas’ disease is a relevant predictor of the inferior quality of health services and is prevalent in areas where individuals are less likely to receive essential treatments, have less access to highly complex services and are more likely to develop severe forms of the disease (6).

Mortality from Chagas’ disease in Ceará represents a major challenge to the management of the Unified Health System, as well as to the socioeconomic development of the state. The situation reinforces stigmas and disparities, reflected in the greater likelihood of occurrence in populations experiencing multifactorial poverty, with low human development and less access to health services (15).

Mortality from Chagas’ disease in Ceará has distinct spatial and temporal patterns, with relevant variations between macroregions. The analyses identified areas of greatest risk, especially in the Litoral Leste and Sertão Central macro-regions, influenced by factors such as income and human development inequality. For future perspectives, the development of population studies is recommended, with individualized and preferably primary data, based on designs with greater methodological accuracy, such as cohort and case-control studies.

## Data Availability

The database used to conduct the analyses is available in the public SciELO Data repository, using the identifier: https://doi.org/10.48331/scielodata.DH2UVY
