# Supplementary material for: Spatial and temporal patterns and factors associated with mortality from Chagas’ disease: an ecological study, Ceará, 2002-2022
Source: Epidemiol Serv Saude. 2025 Aug 8;34:e20240852. doi: 10.1590/S2237-96222025v34e20240852.en (PMC12342723; doi:10.1590/S2237-96222025v34e20240852.en)
Supplement: Translation

# Translation: Padrões espaciais e temporais e fatores associados à mortalidade por doença de Chagas: estudo ecológico, Ceará, 2002-2022

**DOI:** 10.1590/S2237-96222025v34e20240852.pt

**Authors:** Lara Lídia Ventura Damasceno, George Jó Bezerra Sousa, Thiago Santos Garces, Virna Ribeiro Feitosa Cestari, Jéssica Cristina Moraes de Araújo, Thereza Maria Magalhães Moreira, Maria Lúcia Duarte Pereira

**Affiliations:** 1 Universidade Estadual do Ceará, Programa de Pós-Graduação Cuidados Clínicos em Enfermagem e Saúde, Fortaleza, CE, Brasil; 2 Ministério da Saúde, Brasília, DF, Brasil; 3 Universidade Estadual do Ceará, Programa de Pós-Graduação em Saúde Coletiva, Fortaleza, CE, Brasil

**Keywords:** Doença de Chagas, Mortalidade, Indicadores Demográficos, Análise Espacial, Estudos Ecológicos

## Abstract

**Objetivo:**

Descrever o padrão temporal e espacial da mortalidade por doença de Chagas no Ceará no período 2002-2022.

**Métodos:**

Trata-se de estudo ecológico, que considerou o Ceará, suas macrorregiões de saúde e municípios como unidades de análise, com base em dados secundários do Sistema de Informação sobre Mortalidade. Realizou-se análise temporal, para o cálculo da variação percentual anual, bem como os indicadores de autocorrelação espacial (índice de Moran global e local, Getis-Ord Gi* e varredura scan). Os indicadores foram inseridos em modelos de regressão não espacial e espacial.

**Resultados:**

Foram notificados 1.041 óbitos, com taxa de mortalidade média de 0,57 óbito/100 mil habitantes. O Cariri (35,45%) e o Litoral Leste (23,05%) destacaram-se nas notificações, enquanto o Litoral Norte resguardou maior taxa de mortalidade (2,15/100 mil habitantes). Houve aumento significativo nas taxas de mortalidade pela doença no Litoral Leste e no Sertão Central. Foram observados coeficientes elevados para os índices de Gini (9,6; p-valor<0,001) e de desenvolvimento humano municipal (17,1; p-valor<0,001), o que sugeriu que áreas com maior desigualdade de renda e menores índices de desenvolvimento humano são impactadas em maior proporção pela mortalidade por doença de Chagas. Áreas de alta mortalidade tendem a estar próximas de outras áreas também com altas taxas de mortalidade.

**Conclusão:**

Identificaram-se áreas de maior risco, especialmente nas macrorregiões Litoral Leste e Sertão Central, enquanto outras regiões apresentaram estabilidade ao longo do período. Os modelos espaciais apontaram influência por fatores como desigualdade de renda e desenvolvimento humano.

**Aspectos éticos**

Esta pesquisa utilizou bancos de dados de domínio público e anonimizados.:

## Introdução

A doença de Chagas afeta de 6 a 7 milhões de pessoas globalmente, ao mesmo tempo que é responsável por 200 mil óbitos anuais (1). O Brasil, país de clima tropical e subtropical, lidera altas taxas de prevalência e mortalidade pela doença no contexto da América Latina. O país é responsável por mais de 10 mil óbitos ao ano, 1 milhão de casos e 25 milhões de pessoas em risco para a infecção, o que reforça o padrão de endemicidade da doença na América Latina. Entre as regiões brasileiras, o Nordeste desempenha relevante papel na epidemiologia nacional da doença de Chagas, dada confluência de fatores morfoclimáticos da caatinga, prevalência de habitações humanas precárias do tipo casa de taipa, com ambientes peridomésticos suscetíveis à presença do vetor, além da predominância de populações em fragilidade social e com limitações no acesso aos cuidados de saúde (2-3).

O Ceará integra uma das áreas prioritárias de atuação do Programa Brasil Saudável, lançado em 2024 pelo Ministério da Saúde, a fim de eliminar doenças determinadas socialmente, incluindo a doença de Chagas (4). Esse rol de doenças afeta desproporcionalmente populações pobres, tecendo estreita relação com aspectos ambientais, sociais e econômicos, ao mesmo tempo que tem sido marcado historicamente por estigma, discriminação e baixa atratividade econômica (5).

Considerando-se as técnicas de georreferenciamento como tecnologia de subsídio à pesquisa, gestão e assistência em saúde, destaca-se a relevância do mapeamento das características espaciais e temporais da doença de Chagas. Ressalta-se também a influência dessa doença na relação saúde-doença das populações, com vistas ao reconhecimento de demandas e construção de evidências para gestão de políticas públicas efetivas, consistentes e sustentáveis, com base na identificação de aglomerados de alto risco (6). 

Este estudo teve como objetivo descrever o padrão temporal e espacial da mortalidade por doença de Chagas no Ceará no período 2002-2022.

## Métodos

### Delineamento

Trata-se de estudo ecológico que utilizou técnicas de geoprocessamento e série temporal. Foram considerados os dados secundários do Sistema de Informação sobre Mortalidade do Departamento de Informação e Informática do Sistema Único de Saúde.

### Contexto

Foram consideradas a área geográfica do Ceará, suas macrorregiões de saúde e municípios como unidades de análise. O estado está localizado na região Nordeste do Brasil e tem como capital Fortaleza. A extensão territorial é de 148.894,447 km^2^ e tem população estimada de 8.794.957 habitantes; destes, estima-se que pelo menos 22,6% residem em domicílios localizados em áreas rurais, dispostos em 184 municípios e cinco macrorregiões de saúde: Fortaleza, Cariri, Litoral Leste, Sobral e Sertão Central. As macrorregiões de saúde são compostas por municípios fronteiriços, que compartilham características econômicas e sociais similares (7).

O Ceará abriga vasta quantidade de espécies de vetores do *Trypanosoma cruzi*, agente etiológico da doença de Chagas. Estes são insetos hematófagos, da subfamília Triatominae, conhecidos popularmente como barbeiros. Acerca das espécies nativas do bioma da caatinga, nas zonas semiáridas, destacam-se: *Triatoma brasiliensis, Triatoma pseudomaculata, Triatoma sordida, Panstrongylus megistus, Panstrongylus lutzi* e *Rhodnius nasutus*, como as de maior relevância epidemiológica (8).

### Fonte de dados

Consideraram-se todos os registros de óbitos notificados no Sistema de Informação sobre Mortalidade entre janeiro de 2002 e dezembro de 2022 tendo como causa base o código B57 da Classificação Internacional de Doenças (CID-10), que inclui: B570 – Forma aguda da doença de Chagas, com comprometimento cardíaco; B571 – Forma aguda da doença de Chagas, sem comprometimento cardíaco; B572 – doença de Chagas (crônica) com comprometimento cardíaco; B573 – doença de Chagas (crônica) com comprometimento do aparelho digestivo; B574 – doença de Chagas (crônica) com comprometimento do sistema nervoso; e B575 – doença de Chagas (crônica) com comprometimento de outros órgãos. Os bancos de dados utilizados para construção dos resultados estão disponíveis no repositório SciELO Data e são de acesso público (9).

### Métodos estatísticos

Para o cálculo da taxa bruta de mortalidade, tomou-se por base o número de óbitos por doença de Chagas por ano no Ceará, dividido pelo denominador da população residente no estado naquele ano, multiplicado pelo coeficiente de 100 mil habitantes. As taxas de mortalidade foram calculadas para as variáveis sexo, faixa etária, raça/cor da pele e escolaridade, considerando o número de óbitos e população estratificadas, multiplicado pelo coeficiente de 100 mil habitantes.

Na análise da série temporal, os dados foram alocados no software Joinpoint Regression Program, que identificou os pontos de inflexão do período, além de calcular a variação percentual anual, a variação percentual anual média e intervalo de confiança de 95% (IC95%). O ano foi definido como variável independente e a taxa de mortalidade para cada ano como variável dependente, assumindo-se de zero a dois pontos de inflexão, segundo a permutação de Monte Carlo. Para todas as análises, considerou-se a autocorrelação de primeira ordem dos erros, ao passo que dados em saúde possuíam dependência de eventos anteriores. Valores da variação percentual anual e da variação percentual anual média negativos ou positivos com significância estatística indicavam séries temporais de padrão decrescente e crescente. Quando não houve significância estatística, a série foi considerada estacionária (10).

Na análise espacial, foi calculada a taxa de mortalidade média para cada município (taxa bruta), adotando com numerador a média de óbitos por doença de Chagas para cada município, dividido pela população do município no meio do período, correspondente ao ano de 2012, multiplicado pelo coeficiente de 100 mil habitantes. Para minimizar instabilidades, as taxas brutas foram suavizadas pelo método bayesiano empírico local, que incluiu os efeitos espaciais dos vizinhos geográficos (municípios limítrofes), a partir da atribuição de valores para municípios vizinhos (1) e para não vizinhos (0), através da matriz de pesos espaciais do tipo convenção rainha (11).

A identificação de aglomerados espaciais deu-se a partir de métodos de autocorrelação global e local, dispostos pelo índice de Moran global e pelo índice de Moran local (Lisa Map), respectivamente, pelo Getis-Ord Gi* e pela varredura scan. O Lisa Map auxiliou a mensuração do grau de associação espacial de cada município, através da identificação de padrões alto-alto, baixo-baixo, alto-baixo e baixo-alto. Os padrões alto-alto e baixo-baixo referiram-se a áreas com valores elevados ou reduzidos para mortalidade e foram agrupados geograficamente, indicando a associação espacial positiva. Os padrões alto-baixo e baixo-alto indicaram associação espacial negativa, ou seja, representaram municípios com valores discrepantes em relação aos seus vizinhos. O Getis-Ord Gi* inferiu aglomeração em áreas com altas taxas (áreas quentes) e áreas com baixas taxas (áreas frias), a partir da criação de escores Z (12).

Foi realizada a varredura scan puramente espacial para identificação de aglomerados e cálculo do risco relativo para cada município, adotando o modelo discreto de Poisson. Consideraram-se os critérios: tamanho máximo do aglomerado igual a 50,0% da população sob risco, aglomerados em formato circular e 999 replicações. Valores de risco relativo >1 foram visualizados em municípios com risco de óbito por doença de Chagas superior ao risco do país (13).

Foram selecionados alguns indicadores no Atlas de Desenvolvimento Humano do Brasil para analisar a influência de indicadores socioeconômicos na mortalidade por doença de Chagas nos municípios do Ceará: índice de Gini, índice de desenvolvimento humano municipal (IDHM), índice de vulnerabilidade social, taxa de analfabetismo em maiores de 18 anos, taxa de desocupação em maiores de 18 anos, percentual de pessoas inscritas no Cadastro Único para Programas Sociais que recebem o Bolsa Família, percentual de pessoas em domicílios com abastecimento de água e esgotamento sanitário inadequados e percentual de pessoas em domicílios com paredes que não sejam de alvenaria ou madeira aparelhada.

Utilizaram-se diferentes métodos de regressão espaciais e não espaciais. Aplicou-se a *generalized linear model* (GLM), devido à variabilidade das taxas de mortalidade e ao não atendimento dos pressupostos da regressão linear simples. Em seguida, foram testados modelos espaciais, como *spatial lag* e *spatial error*, para capturar a autocorrelação espacial dos dados, avaliados por meio dos parâmetros Rho (*spatial lag*) e Lambda (*spatial error*). O ajuste desses modelos foi comparado utilizando o critério de informação de Akaike, com menores valores indicando melhor qualidade do ajuste. Por fim, o modelo *geographically weighted regression* (GWR) foi ajustado para identificar variações locais nos coeficientes. A qualidade da análise foi mensurada pelo pseudo R^2^, que refletiu a proporção de variabilidade explicada localmente. Todas as análises de regressão foram realizadas no software R (versão 4.4.1) com os pacotes spdep, GWmodel, sf e spgwr.

Os dados, após extraídos em formato CSV, foram tabulados em planilhas do software Microsoft Excel e importados para o software QGis, no qual todos os mapas foram confeccionados. A estatística bayesiana, o teste de autocorrelação espacial e a técnica Getis-Ord Gi* foram executados no software GeoDa. A técnica de varredura e cálculo do RR deram-se a partir do software SaTScan. Todas as regressões com indicadores de desenvolvimento social foram executadas no software R.

## Resultados

Foram notificados 1.041 óbitos por doença de Chagas no Ceará no período 2002-2022, equivalente à média de 50 óbitos/ano. As maiores taxas de mortalidade foram registradas em homens, de 80 anos ou mais, de raça/cor da pele parda, analfabetos ou com menos de três anos de estudo (Tabela 1).

**Tabela 1:** Caracterização sociodemográfica dos óbitos e taxa de mortalidade padronizada para doença de Chagas no período 2002-2022. Ceará, 2024 (n=1.041)

Variável	Frequência	Taxa de mortalidade^a^
Sexo		
Masculino	694	0,78
Feminino	347	0,37
**Faixa etária** (anos)		
0-4	1	0,01
5-9		
10-14		
15-19	1	0,01
20-29	6	0,02
30-39	41	0,15
40-49	112	0,50
50-59	206	1,31
60-69	230	2,16
70-79	224	3,54
80+	219	6,64
**Raça/cor da pele**		
Branca	265	0,51
Preta	53	0,42
Amarela	3	1,27
Parda	676	0,57
Indígena	2	0,24
**Escolaridade** (anos)		
Nenhuma	350	0,19
1-3	302	0,16
4-7	144	0,08
8-11	46	0,03
12+	14	0,01

^a^Taxa de mortalidade calculada para o coeficiente de 100 mil habitantes.

A taxa de mortalidade do estado foi 0,57 óbitos/100 mil habitantes. As notificações foram realizadas, majoritariamente, nas macrorregiões de saúde Cariri (n=369; 35,35%; 1,21 óbitos/100 mil habitantes) e Litoral Leste (n=240; 23,05%; 2,15 óbitos/100 mil habitantes). As demais macrorregiões foram Fortaleza (n=182; 17,48%; 0,19 óbito/100 mil habitantes), Sobral (n=172; 16,52%; 0,51 óbito/100 mil habitantes) e Sertão Central (n=78; 7,49%; 0,59 óbito/100 mil habitantes).

A série temporal indicou tendência estacionária para o Ceará e nas macrorregiões Cariri, Sobral e Fortaleza. O Litoral Leste apresentou crescimento anual significativo de 2,03% (IC95% 0,11; 4,^19^). A macrorregião Sertão Central mostrou aumento expressivo de 8,62% entre 2002 e 2020 (IC95% 4,89; 25,08), quando passou a adotar tendência estacionária (Tabela 2).

**Tabela 2:** Variação percentual anual, variação percentual anual média, ponto de inflexão e intervalo de confiança de 95% (IC95%) das taxas de mortalidade por doença de Chagas no período 2002-2022. Ceará, 2024 (n=1.041)

	Variação percentual anual 1 (IC95%)	Ponto de inflexão	p-valor	Variação percentual anual 2 (IC95%)	Variação percentual anual média (IC95%)	p-valor
Ceará	0,90 (-0,19; 2,07)				0,90 (-0,19; 2,07)	
**Litoral Leste**/Jaguaribe	2,03^a^ (0,11; 4,19)		0,042		2,03^a^ (0,11; 4,19)	0,042
**Sertão Central**	8,62^a^ (4,89; 25,08)	2020	0,026	-58,51 (-81,73; 2,28)	-1,34 (-8,74; 7,42)	
Cariri	0,27 (-1,63; 2,31)				0,27 (-1,63; 2,31)	
Sobral	-0,19 (-2,73; 2,39)				-0,19 (-2,73; 2,39)	
Fortaleza	0,03 (-2,67; 2,90)				0,03 (-2,67; 2,90)	

As análises espaciais confirmaram a consistência dos resultados obtidos por diferentes métodos, incluindo o índice de Moran global (0,254), que indicou autocorrelação espacial positiva. Notaram-se taxas brutas de mortalidade distribuídas irregularmente ao longo do período (Figura 1A). Quando suavizadas pelo método bayesiano empírico local (Figura 1B), as taxas mais elevadas foram observadas nos municípios do Litoral Leste e Cariri. 

**Figura 1:** Distribuição espacial das taxas de mortalidade bruta por doença de Chagas (A), taxas suavizadas pelo método bayesiano empírico local (B), indicador local de autocorrelação espacial (C), significância estatística respectiva (D), indicador local de autocorrelação Getis-Ord Gi* (E), significância estatística respectiva (F), aglomerados puramente espaciais identificados por varredura scan (G) e risco relativo municipal (H), no período 2002-2022. Ceará, 2024 (n=1.041)

Padrões alto-alto (Figura 1C), ou seja, municípios com altas taxas de mortalidade rodeados por outros com taxas igualmente altas, foram visualizados nas localidades de Russas, Palhano, Itaiçaba, Jaguaruana, Alto Santo, Iracema e Potiretama no Litoral Leste; em Lavras da Mangabeira no Cariri; e em Novo Oriente na região de Sobral. A faixa litorânea do estado que compreende as macrorregiões Sobral e Fortaleza apresentaram padrão baixo-baixo, que representaram, de maneira geral, localidades com baixas taxas de mortalidade para doença de Chagas. 

Padrões alto-baixo puderam ser visualizados em Aracati, Jaguaribe e Pereiro no Litoral Leste; em Iguatu, Umari e Ipaumirim na região do Cariri; e em Solonópole no Sertão Central. Apenas Alcântaras foi visualizado em padrão baixo-alto, localizado na região do Cariri. Ambos os padrões revelaram localidades com valores diferentes em relação a seus vizinhos, o que representou discrepâncias espaciais. A técnica Getis-Ord Gi* confirmou áreas quentes nas regiões Litoral Leste e Cariri e áreas frias em Sobral e Fortaleza (Figura 1E). A varredura scan detectou seis aglomerados espaciais (Figura 1G), sendo Iracema, município do Litoral Leste, o aglomerado primário.

Observaram-se os modelos de regressão aplicados aos indicadores de desenvolvimento social associados à mortalidade por doença de Chagas no Ceará (Tabela 3). O modelo GLM apresentou o pior ajuste, o que indicou que a variação espacial dos dados não foi bem capturada. O modelo *spatial lag* obteve melhor adequação aos dados, o que comprovou que a inclusão de estrutura espacial foi fundamental para capturar a dependência espacial entre os municípios. O coeficiente de Gini nesse modelo refletiu que o efeito da desigualdade de renda é menos acentuado quando considerada a dependência espacial. O IDHM apresentou coeficiente próximo ao nível de significância. O modelo *spatial error* indicou correlação espacial nos resíduos, sugerindo que, embora o ajuste seja bom, havia dependências não capturadas pelo modelo. O modelo GWR apresentou ajuste inferior aos outros modelos espaciais, o que indicou que os efeitos das variáveis alteravam espacialmente de maneira não uniforme.

**Tabela 3:** Associação entre indicadores de desenvolvimento social e mortalidade por doença de Chagas, segundo modelos de regressão *generalized linear model* (GLM), *spatial lag*, *spatial error* e *geographically weighted regression* (GWR), no período 2002-2022. Ceará, 2024 (n=1.041)

Indicador	GLM		Spatial error			Spatial lag			GWR	
	Coeficiente	EP^a^	Coeficiente	EP^a^	p-valor	Coeficiente	EP^a^	p-valor	Coeficiente	EP^a^
Constante	16,63	<0,001	8,11	3,88	0,036	8,05	3,41	0,018	13,67	3,89
Gini	9,64	5,08	4,43	1,91	0,021	4,51	1,61	0,005	5,99	5,972
IDHM^b^	17,19	<0,001	6,78	4,42	0,125	7,41	3,92	0,059	8,43	14,60
IVS^c^	0,14	0,94	-	-	-	-	-	-	-	-
Taxa de analfabetismo	<0,001	0,65	-	-	-	-	-	-	-	-
Taxa de desocupação em maiores de 18 anos	0,03	0,28	-	-	-	-	-	-	-	-
Bolsa Família	<0,001	0,80	-	-	-	-	-	-	-	-
Domicílios sem água ou esgotamento sanitário	<0,001	0,67	-	-	-	-	-	-	-	-
Domicílios sem parede de alvenaria ou madeira aparelhada	0,01	0,30	-	-	-	-	-	-	-	-
Rho (*spatial lag*)^d^	-	-	-	-	-	0,55	0,07	1,181	-	-
Lambda (*spatial error*)^e^	-	-	0,55	0,07	1,571	-	-	-	-	-
R^2f^	0,14	-	0,32	-	-	0,32	-	-	0,09	-
Critério de informação de Akaike^g^	540,72	-	500,29	-	-	497,26	-	-	528,17	-

^a^EP=erro-padrão; ^b^IDHM=índice de desenvolvimento humano municipal; ^c^IVS=índice de vulnerabilidade social; ^d^Mede a dependência espacial na variável dependente e indica o efeito da unidade sobre suas vizinhas; ^e^Avalia a correlação espacial nos resíduos do modelo, indicando dependência não capturada pela regressão tradicional; ^f^Representa a proporção da variância explicada pelo modelo, indicando seu ajuste aos dados. ^g^Mede a qualidade do modelo considerando ajuste e complexidade, sendo menor para modelos mais eficientes.

Apontou-se a distribuição espacial dos coeficientes estimados pelo GWR para os indicadores de desenvolvimento social (Figura 2 e Tabela 3). Regiões como Litoral Leste e Cariri apresentaram coeficientes negativos mais acentuados para o Gini, indicando maior influência da desigualdade de renda na redução das taxas de mortalidade localmente, enquanto áreas como Sobral e Fortaleza mostraram efeitos menores e não significativos. Para o IDHM, as regiões Litoral Norte e Sertão Central tiveram coeficientes positivos e significativos, o que mostrou maior impacto do desenvolvimento humano sobre a mortalidade. Esses padrões reforçaram a superioridade dos modelos *spatial lag* e *spatial error* na captura da dependência espacial global e justificaram a utilidade do GWR para detectar variações locais, ao evidenciar a complexidade espacial desses determinantes na mortalidade por doença complementando a análise global de Chagas.

**Figura 2:** Regressão espacial geograficamente ponderada da distribuição espacial da taxa de mortalidade por doença de Chagas para o índice de desenvolvimento humano (A), significância estatística respectiva (B), distribuição espacial da taxa de mortalidade por doença de Chagas para o índice de Gini (C), significância estatística respectiva (D), no período 2002-2022. Ceará, 2024 (n=1.041)

## Discussão

A mortalidade por doença de Chagas no Ceará manteve-se estável, com perfil de prevalência entre homens, idosos a partir de 80 anos, de raça/cor da pele parda e amarela e com baixa escolaridade. A maior ocorrência foi nas macrorregiões Litoral Leste e Cariri, enquanto Fortaleza e Sobral apresentaram menor impacto. A relação entre indicadores socioeconômicos e mortalidade por doença de Chagas variou regionalmente e evidenciou a desigualdade de renda e o desenvolvimento humano municipal como fatores de maior influência. Esses achados reforçaram a persistência da doença de Chagas como problema de saúde pública no estado, especialmente em áreas historicamente vulneráveis.

Como limitação deste estudo, ressalta-se o uso de dados secundários, pois foram imprecisos em seus registros, decorrentes de preenchimento inadequado do sistema, além das subnotificações. Os profissionais envolvidos e responsáveis no processo de notificação dos óbitos por doença de Chagas foram: médicos, enfermeiros, profissionais do Serviço de Verificação de Óbito e do Instituto Médico-Legal, epidemiologistas e técnicos da vigilância em saúde e profissionais do Sistema de Informação sobre Mortalidade. Os resultados da análise devem ser interpretados com a devida cautela, levando em consideração potenciais vieses ou imprecisões dos dados.

O perfil epidemiológico e a localização geográfica do evento favoreceram a interpretação de que a infecção chagásica estava relacionada a piores desfechos, como a mortalidade, em residentes e trabalhadores de áreas rurais, teoricamente mais expostos ao agente etiológico da doença (2).

Apesar da predominância de casos em zona rural, ressalta-se que as macrorregiões possuem outras atividades econômicas, como o comércio e o artesanato. A doença de Chagas e seus desfechos, inicialmente restritos às áreas rurais e menos populosas, demonstraram tendência de urbanização, favorecendo a transmissão da doença (14-15). Estes achados foram semelhantes aos dados secundários sobre mortalidade por doenças negligenciadas no Brasil, incluindo a doença de Chagas (16).

O perfil étnico identificado demonstrou associação significativa entre a raça/cor da pele parda e negra e o pior prognóstico de doença de Chagas. Esse resultado deve ser analisado com cautela, pois a população nordestina apresenta elevado grau de miscigenação, o que dificulta classificação precisa.

Os achados revelaram que a mortalidade por doença de Chagas no Ceará está associada, de maneira direta e inversamente proporcional, ao índice de Gini e ao IDHM, respectivamente, ou seja, quanto maior a desigualdade de renda e piores condições de vida e desenvolvimento, maior a taxa de mortalidade por doença de Chagas. O cenário deve-se à considerável heterogeneidade socioeconômica da região Nordeste brasileira, reconhecida como a mais pobre do país. Essas desigualdades se fazem presentes também no componente de serviços/assistência à saúde no acesso e na continuidade do cuidado, principalmente em macro e microrregiões com elevada vulnerabilidade, como o Sertão Central e o Litoral Leste (18). 

Tais regiões são caracterizadas por possuir concentração de municípios de extrema pobreza, representados por baixos IDHM e índice brasileiro de privação e acesso à saúde. A desigualdade social influencia a busca por melhores condições de vida e, em vista disso, muitos indivíduos se deslocam para outras regiões e estados. Frequentemente, permanecem com baixo padrão de vida, aglomerando-se em territórios excluídos e carentes de serviços públicos, incluindo a atenção à saúde.

O Litoral Leste é marcado pelo vazio assistencial no âmbito da alta e média complexidade, que motivou o investimento no Hospital Regional Vale do Jaguaribe, inaugurado no final de 2021 (19). Municípios como Russas e Jaguaruana, apesar de polos comerciais densamente populosos, exibem fragilidades não superadas no que tange à disponibilidade e à viabilidade de cuidados em saúde e estratégias de combate à pobreza. As fragilidades associaram-se a características geográficas, de transporte, inadequada delimitação da área de abrangência dos serviços, falta de apoio governamental e distribuição de recursos deficitária (20-21). 

A região do Cariri detinha o contingente de mais de 400 mil pessoas em situação de extrema pobreza e concentrava dispositivos de alta complexidade em Barbalha, em Brejo Santo, no Crato e em Juazeiro do Norte, no extremo sul do estado, tendo como referência o Hospital Regional do Cariri, inaugurado em 2011. As autoridades de saúde da região, em cooperação com a Organização Pan-Americana da Saúde, lideram o projeto “De braços abertos”, com enfoque na descentralização e regionalização das intervenções de saúde, visando à planificação e ao reforço das linhas de cuidado (22).

A região Norte, detentora de padrões baixo-baixo, tem como sede Sobral, e é reconhecida pela situação econômica bem consolidada, além do polo educacional, com sistema de ensino consistentemente classificado entre os melhores do Ceará e do Brasil. É responsável pela maior densidade de serviços de saúde da região, com destaque ao Hospital Regional Norte, à Santa Casa de Sobral e ao Hospital do Coração. Estes são referências em média e alta complexidade e desempenham papel central na assistência de saúde da região, atuando como principal destino para referência de urgências, emergências e procedimentos eletivos (23). 

Postula-se que o óbito por doença de Chagas é relevante preditor da inferioridade da qualidade dos serviços de saúde e é prevalente em áreas onde os indivíduos possuem menor probabilidade de receber tratamentos essenciais, menor acesso a serviços de alta complexidade e maior probabilidade de desenvolver formas graves da doença (6).

A mortalidade por doença de Chagas no Ceará representa amplo desafio à gestão do Sistema Único de Saúde, bem como ao desenvolvimento socioeconômico do estado. A situação reforça estigmas e disparidades, traduzidas a partir da maior probabilidade de ocorrência em populações em pobreza multifatorial, com baixo desenvolvimento humano e menor acesso aos serviços de saúde (15).

A mortalidade por doença de Chagas no Ceará possui padrões espaciais e temporais distintos, com variações relevantes entre as macrorregiões. As análises identificaram áreas de maior risco, especialmente nas macrorregiões Litoral Leste e Sertão Central, sendo influenciada por fatores como desigualdade de renda e desenvolvimento humano. Para futuras perspectivas, considera-se o desenvolvimento de estudos populacionais, com dados individualizados, de preferência primários, tomando por base delineamentos de maior acurácia metodológica, como estudos de coorte e caso-controle.

## Data Availability

O banco de dados utilizado para a condução das análises está disponível no repositório SciELO Data, de acesso público, mediante identificador: https://doi.org/10.48331/scielodata.DH2UVY
